# Evaluation of the effectiveness and safety of adding ivermectin to treatment in severe COVID-19 patients

**DOI:** 10.1186/s12879-021-06104-9

**Published:** 2021-05-04

**Authors:** Nurullah Okumuş, Neşe Demirtürk, Rıza Aytaç Çetinkaya, Rahmet Güner, İsmail Yaşar Avcı, Semiha Orhan, Petek Konya, Bengü Şaylan, Ayşegül Karalezli, Levent Yamanel, Bircan Kayaaslan, Gülden Yılmaz, Ümit Savaşçı, Fatma Eser, Gürhan Taşkın

**Affiliations:** 1 Afyonkarahisar Health Sciences University, Afyonkarahisar, Turkey; 2 Haydarpasa Sultan Abdulhamid Han Training and Research Hospital, Istanbul, Turkey; 3 Ankara Yıldırım Beyazıt University, Ankara City Hospital, Ankara, Turkey; 4 Gulhane Faculty of Medicine, University of Health Sciences, Ankara, Turkey

**Keywords:** SARS CoV-2, COVID-19, Ivermectin, Treatment

## Abstract

**Background and objectives:**

An effective treatment option is not yet available for SARS-CoV2, which causes the COVID-19 pandemic and whose effects are felt more and more every day. Ivermectin is among the drugs whose effectiveness in treatment has been investigated. In this study; it was aimed to investigate the presence of gene mutations that alter ivermectin metabolism and cause toxic effects in patients with severe COVID-19 pneumonia, and to evaluate the effectiveness and safety of ivermectin use in the treatment of patients without mutation.

**Materials and methods:**

Patients with severe COVID19 pneumonia were included in the study, which was planned as a prospective, randomized, controlled, single-blind phase 3 study. Two groups, the study group and the control group, took part in the study. Ivermectin 200 mcg/kg/day for 5 days in the form of a solution prepared for enteral use added to the reference treatment protocol -hydroxychloroquine + favipiravir + azithromycin- of patients included in the study group. Patients in the control group were given only reference treatment with 3 other drugs without ivermectin. The presence of mutations was investigated by performing sequence analysis in the *mdr1/abcab1* gene with the Sanger method in patients included in the study group according to randomization. Patients with mutations were excluded from the study and ivermectin treatment was not continued. Patients were followed for 5 days after treatment. At the end of the treatment and follow-up period, clinical response and changes in laboratory parameters were evaluated.

**Results:**

A total of 66 patients, 36 in the study group and 30 in the control group were included in the study. Mutations affecting ivermectin metabolism was detected in genetic tests of six (16.7%) patients in the study group and they were excluded from the study. At the end of the 5-day follow-up period, the rate of clinical improvement was 73.3% (22/30) in the study group and was 53.3% (16/30) in the control group (*p = 0.10*). At the end of the study, mortality developed in 6 patients (20%) in the study group and in 9 (30%) patients in the control group (*p = 0.37*). At the end of the follow-up period, the average peripheral capillary oxygen saturation (SpO2) values of the study and control groups were found to be 93.5 and 93.0%, respectively. Partial pressure of oxygen (PaO2)/FiO2 ratios were determined as 236.3 ± 85.7 and 220.8 ± 127.3 in the study and control groups, respectively. While the blood lymphocyte count was higher in the study group compared to the control group (1698 ± 1438 and 1256 ± 710, respectively) at the end of the follow-up period (*p = 0.24*); reduction in serum C-reactive protein (CRP), ferritin and D-dimer levels was more pronounced in the study group (*p = 0.02, p = 0.005 and p = 0.03*, respectively).

**Conclusions:**

According to the findings obtained, ivermectin can provide an increase in clinical recovery, improvement in prognostic laboratory parameters and a decrease in mortality rates even when used in patients with severe COVID-19. Consequently, ivermectin should be considered as an alternative drug that can be used in the treatment of COVID-19 disease or as an additional option to existing protocols.

**Supplementary Information:**

The online version contains supplementary material available at 10.1186/s12879-021-06104-9.

## Introduction

Since the first case was reported in Wuhan, China in December 2019, the Severe Acute Respiratory Syndrome Coronavirus 2 (SARS CoV-2) induced COVID-19 outbreak which has surrounded the whole world with great speed, still continues its effect as a pandemic. According to current data, more than 66 million people around the world have been affected by the epidemic and more than 1.500.000 people have died due to Coronavirus Disease 2019 (COVID-19) [1, 2].

Many drugs with antiviral, anti-inflammatory and immunomodulatory properties that are currently used in the treatment of COVID-19, unfortunately cannot provide a complete cure [3]. One of the drugs whose effectiveness has been investigated in the treatment of COVID-19 is ivermectin, a drug from the avermectin family which is produced semisynthetically in the structure of *22,23 dihydroavermectin B1*. Ivermectin is used effectively in the treatment of human parasitosis such as ascariasis, cutaneous larva migrans, strongyloidiasis, onchocerciasis, and scabies and it’s oral use is also approved by the U.S. Food and Drug Administration (FDA) [4]. In addition to its antiparasitic activity, in vitro studies have shown that it has also antiviral activity against many viruses such as human immunodeficiency virus (HIV-1), dengue virus and west nile virus (WNV) [5].

In a recent in vitro study published in Australia, the efficacy of ivermectin on SARS CoV-2 was evaluated; the Vero/hSLAM cells infected with SARS CoV-2 in vitro were exposed to ivermectin, and it was reported that there was a 99.8% reduction in viral load 48 h later [6].

It has been reported that ivermectin may have antiviral effects by inhibiting the importin (IMP) a/b1 receptor, which is responsible for transmitting viral proteins into the host cell nucleus [6]. Based on these data, it has been suggested that ivermectin may also affect SARS-CoV-2 replication through IMP a/b1 inhibition [7]. When all these data were evaluated, it was thought that ivermectin may also be effective in COVID-19 patients.

One of the major limitations in the use of ivermectin is the possible side effects of the drug on the central nervous system (CNS). The most common side effects during ivermectin treatment have been reported as fever, headache, dizziness, pruritus and rash, but neurological side effects such as encephalopathy, confusion and coma have also been reported during its use for the treatment of onchocerciasis. It has been stated that these serious neurological adverse events after ivermectin therapy may be related to CYP3A4 gene inhibition or polymorphisms in the MDR-1/ABCB1 gene [8].

It is known in the literature that MDR-1/ABCB1 gene products control ivermectin entry into the barrier cells of gastrointestinal system and CNS in some animals and parasites and act as a carrier molecule. Similarly, it has also been reported that haplotypes and mutations of the CYP3A4 gene, which encodes a carrier molecule, cause toxic effects or drug dose deficiency by changing the metabolic rate of ivermectin [9].

In this study; it was mainly aimed to investigate the effectiveness of adding ivermectin to the treatment in patients with severe COVID-19 pneumonia. In addition, it was also aimed to investigate the presence of genes that alter ivermectin metabolism and cause toxic effects in patients included in the study and to investigate the safety of ivermectin in patients with and without mutations.

## Material and methods

This prospective, randomized, controlled, single-blind phase 3 multicenter clinical trial (conducted between May–September 2020) assessed the effectiveness and safety of ivermectin use in the treatment of patients without mutation. Patients who were hospitalised with a pre-diagnosis of severe pneumonia* and thereafter diagnosis of COVID-19 was also confirmed microbiologically with Polymerase Chain Reaction (PCR) positivity in respiratory tract samples were included into the study (https://COVID-19bilgi.saglik.gov.tr/depo/rehberler/COVID-19_Rehberi.pdf).

*Patients with at least one of the criteria below were accepted as patients with severe pneumonia and they were randomized to the study and control group, respectively.

- *Presence of tachypnea ≥ 30/min, peripheral capillary oxygen saturation (SpO2) level < 90% in room air, Partial pressure of oxygen (PaO2)/FiO2 < 300 in oxygen receiving patient*
- *Presence of specific radiological finding for Covid-19 in lung tomography (bilateral lobular, peripherally located, diffuse patchy ground glass opacities)*
- *Mechanical ventilation requirement*
- *Acute organ dysfunction findings; patients with SOFA (sepsis-related organ failure assessment) score > 2*

Exclusion criteria included: Children < 18 years old, pregnancy, active breast feeding, concurrent autoimmune disease, chronic liver or kidney disease, immunosuppression, SNP mutation in MDR-1/ABCB1 gene and/or haplotypes and mutations of the CYP3A4 gene.

### Genetic examination

In the patients included in the study group according to randomization, haplotypes and mutations that cause the function losing were investigated by performing sequence analysis of MDR-1/ABCB1 and CYP3A4 genes with Sanger method. Mutation screening was done when the first dose of the research drug ivermectin was given, ivermectin treatment was not continued in patients with mutations detected as a result of genetic examination and these patients were excluded from the study.

### Study design

The study took place from May 2020 September 2020 at four different tertiary referred Research and Education Hospital in Turkey. Study was submitted to Clinical Trials (Clinicaltrials.gov NCT04646109, 27/11/2020) and performed in accordance with the Declaration of Helsinki with the relevant guidelines and regulations. Ethics board approval (Afyonkarahisar Health Science University, Local Ethical Commitee 03.04.2020/139) was attained prior to the commencement of this study. In patients meeting the inclusion criteria, the distinction between study and control groups was made by a single-blind randomized method. Starting from the first patient included in the study, patients with odd numbers were grouped as the study group, and patients with even numbers as the control group.

All participants provided informed consent prior to study enrollment and following informed consent eligible patients underwent standardized symptom questionnaire and physical examination. Additionally, complete blood count test, biochemical blood tests, first SARS CoV-2 PCR results and thoracic tomography findings were recorded.

The reference treatment recommended in the “COVID-19 (SARS CoV-2 Infection) guide” (https://COVID-19bilgi.saglik.gov.tr/depo/rehberler/COVID-19_Rehberi.pdf) prepared by the Turkish Ministry of Health, consisting of hydroxychloroquine *(2x400mg loading dose followed by 2x200mg, po, 5 days)*, favipiravir (*2x1600mg loading dose followed by 2x600mg maintenance dose, po, total 5 days)* and azithromycin *(500 mg first day loading dose, followed by 250 mg/day, po, total 5 days)* (HFA), was applied to all patients in the control and study group.

In addition to the reference treatment, the patients in the study group received ivermectin treatment in the form of a solution prepared for enteral use at 200 microgr/kg/day (9 mg between 36 and 50 kg, 12 mg between 51 and 65 kg, 15 mg between 66 and 79 kg and 200 microgram/kg in > 80 kg) for 5 days (Ivermectin 5 mg/5 ml solution was manufactured by NEUTEC™ Pharmaceutical Company-Turkey, under “Good Manufacturing Practices” (GMP) certification conditions).

During the study; respiratory findings and laboratory parameters of the patients were recorded on the 1st, 3rd and 5th days of the treatment and on the 1st, 3rd and 5th days after the treatment, during the follow-up period. Side effects observed during the treatment in all patients were recorded.

Primary and secondary endpoints for efficacy and safety assessment in the study were determined as follows:

#### Primary endpoint

Clinical responses and drug side effects obtained in patients on the 5th day, at the end of the ivermectin treatment were evaluated. Extubation in mechanically ventilated patients, respiratory rate < 26, SpO2 level in room air > 90%, PaO2 / FiO2 > 300 in patients receiving oxygen, presence of at least two of the 2-point reduction criteria in SOFA (Sequential Organ Failure Assessment) score were evaluated as “*clinical response*”.

#### Secondary endpoint

Clinical responses and drug side effects obtained in patients on the 5th day after the end of ivermectin treatment (Totally 10th day) were evaluated. For clinical response, the presence of at least two of the following criteria was sought: Respiration rate between 22 and 24, SpO2 level in room air > 95%, absence of oxygen requirement and no need for intensive care.

In order to evaluate the treatment response in patients; blood lymphocyte count, C-reactive protein (CRP), ferritin and D-dimer values, changes in polymorphonuclear leukocyte/lymphocyte (PNL/L) ratio, changes in SpO2 value and PaO2/FiO2 ratio were determined and compared between both groups at the primary and secondary endpoints. PCR negativity and mortality rates at the end of the follow-up period were also evaluated in both groups.

### Statistical evaluation

The sample size of the study with an α error of 0.05, a power of 0.95 and a medium effect size of 0.26 according to standardized size effects, was calculated for a 1:1 randomization in 30 patients in the IVM group and 30 in the control group, to detect differences between 2 independent groups in the change in mean viral load in nasopharyngeal swabs among repeated measures. Power analysis was performed with Gpower computer program version 3.1 for Windows. For the quantitative variables whose values were measured repeatedly, Friedman test was used for in-group comparisons, Mann-Whitney U test was used for comparison of control and study groups, and chi-square test was used for categorical variables. For statistical significance, the value of *p* = 0.05 was taken into account and the analyzes were carried out in the SPSS 20.0 package program.

## Results

A total of 66 patients, 36 in the study group and 30 in the control group were included in the study. Six (16.7%) patients in the study group were excluded from the study, continuing only the reference treatment after taking the first dose of ivermectin, as a mutation was detected in genetic tests affecting ivermectin metabolism. When the demographic data and the pre-treatment clinical and laboratory findings of the patients were compared, with no significant difference was found between the study group and the control group in any parameter. Demographic data and pre-treatment clinical and radiological findings of both groups are shown in Table 1.

**Table 1:** Demographic and Clinical Characteristics at Baseline

Patient characteristics	Study group, ***n*** = 30	Control group, ***n*** = 30	***P***
**Gender (male), n (%)**	21 (70)	19 (63.3)	*0.58*
**Age (years) (mean)**	58.17 ± 11.52	66.23 ± 13.31	*0.15*
**Comorbid conditions, n (%)**			
Diabetes mellitus	9 (30)	10 (33.3)	*0.78*
Hypertension	15 (50)	12 (40)	*0.43*
Coronary artery disease	5 (16.7)	8 (26.7)	*0.34*
Cardiac failure	–	1 (3.3)	*0.31*
Chronic Obstructive Pulmonary Disease	6 (20)	3 (10)	*0.27*
Malignancy	–	1 (3.3)	*0.36*
Immunodeficiency	–	1 (3.3)	*0.31*
**Symptoms, n (%)**			
Fever	15 (59)	13 (43.3)	*0.60*
Cough	16 (53.3)	14 (46.7)	*0.60*
Sore throat	3 (10)	1 (3.3)	*0.30*
Dyspnea	23 (76.7)	19 (63.3)	*0.26*
Headache	5 (16.7)	2 (6.7)	*0.22*
Weakness	13 (43.3)	11 (3.7)	*0.59*
Myalgia	9 (30)	7 (23.3)	*0.55*
Diarrhea	1 (3.3)	–	*0.31*
Nausea or vomiting	1 (3.3)	–	*0.31*
**Signs (mean)**			
Body temperature (^0^C)	36.9 ± 0.7	36.8 ± 0.8	*0.15*
Heart rate (per minute)	88 ± 12	92 ± 18	*0.47*
Respiratory rate (per minute)	24 ± 5	24.7 ± 0.7	*0.92*
Systolic pressure (mmHg)	124.39 ± 15.60	124.61 ± 15.37	*0.85*
Diastolic pressure (mmHg)	75.64 ± 9.79	73.43 ± 8.47	*0.07*
Inspiratory ral, n (%)	11 (36.7)	19 (63.3)	*0.03*
SOFA score (mean)	3.12 ± 1.9	2.83 ± 2.1	*0.36*
Mechanic ventilation requirement, n (%)	1 (3.3)	1 (3.3)	*0.98*
**Typical radiological findings, n (%)**	29 (96.7)	27 (90)	*0.30*

### Clinical improvement and mortality

At the end of five-day treatment period (primary endpoint), the rate of clinical improvement was 46.7% (14/30) in the study group and was 36.7% (11/30) in the control group. Similarly at the end of the 5-day follow-up period (secondary endpoint), the rate of clinical improvement was 73.3% (22/30) in the study group and was 53.3% (16/30) in the control group, but the differences were not statistically significant (*p = 0.43 and p = 0.10 respectively*) (Supplementary Table 1).

Patients who died in both groups were recorded until the study was completed (an average of 3 months) and mortality developed in 6 patients (20%) in the study group and in 9 (30%) patients in the control group (*p = 0.37*). When the mean SOFA scores before treatment and at the end of the follow-up period were compared, a significant decrease was found in the study group (*p = 0.009*), while an increase was found in the control group (*p = 0.88*). When the SOFA scores of both groups were compared at the end of the follow-up period, no significant difference was found between them (*p = 0.50*).

At the end of the follow-up period, 16 (57.1%) patients in the study group and 8 (26.7%) in the control group were investigated by PCR test for SARSCoV-2. Of these patients, 14 (87.5%) patients in the study group and 3 (37.5%) patients in the control group were found to become negative and the difference was significantly higher in the study group than in the control group (*p = 0.01*).

### Oxygenation changes

**Fig. 1:**
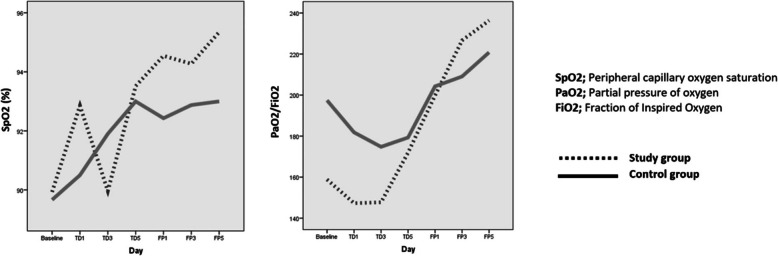
Graphical representation of the change in SpO2 values and PaO2/FiO2 ratios

In both groups, SpO2 values (from 89.9 ± 6.5 to 93.5 ± 4.4% and from 89.7 ± 5.1 to 93.0 ± 3.3% in the study and control groups) ​​were found to be statistically significantly (*p = 0.005* for the study group, *p = 0.003* for the control group) higher at the end of the treatment period compared to the baseline levels. SpO2 levels continued to increase during the follow-up period in both groups. However, at the end of the follow-up period (95.4 ± 2.7% and 93.0 ± 3.9% in the study and control groups), the increase in SpO2 in the study group was statistically significantly higher than in the control group (*p = 0.032*) (Fig. 1).

At the beginning of the treatment period, a decrease was observed in the PaO2/FiO2 ratios in both groups, and then it was observed that the PaO2/FiO2 ratios started to increase. Despite this, it was observed that the increase in the control group was not sufficient at the end of the treatment period and remained slightly below the baseline values (from 197.4 ± 102.3 to 180.1 ± 95.4), while a significant increase was observed in PaO2/FiO2 ratios in the study group compared to the initial values (from 158.8 ± 88.2 to 178.9 ± 98.2 and *p = 0.00*). Increase in PaO2/FiO2 ratios continued in both groups during the follow-up period and at the end of the follow-up period​​ (236.3 ± 85.7 and 220.8 ± 127.3 in the study and control group), the increase in the study group according to the baseline values was again found to be statistically significant (*p = 0.01*). At the end of the follow-up period, although the PaO2 / FiO2 ratios of the study group were higher than the control group, the difference was not significant (*p = 0.39*) (Fig. 1).

### Laboratory parameter changes

#### Blood lymphocyte counts (cell/mm^3^)

**Fig. 2:**
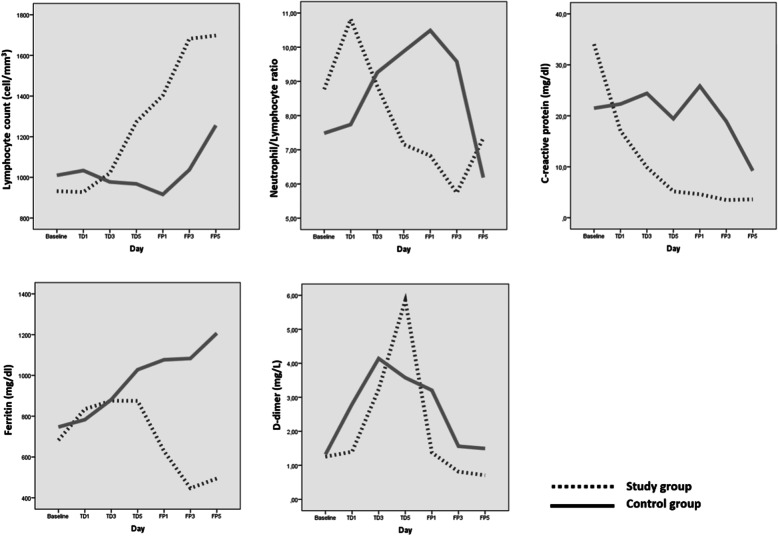
Graphical representation of the change in laboratory parameters

At the end of treatment period, Blood lymphocyte counts increased in the study group and slightly decreased in the control group. The increase in the study group was statistically significant (*p = 0.010*). At the end of the follow-up period, there was an increase in both groups compared to baseline values, and the increase in both groups was statistically significant (*p = 0.008* and *p = 0.05*). When the both groups were compared, no difference was found (*p = 0.24*) (Fig. 2).

#### Polymorphonuclear Leucocyte to Lymphocyte ratios (PNL/L)

During the treatment period, PNL/L ratios decreased in the study group and increased in the control group (*p > 0.05* for two groups). During the follow-up period, PNL/L ratios in both groups decreased and fell below the baseline values. However, this decline in both groups (*p > 0.05* for two groups) and the difference between the two groups at the end of the follow-up period was not significant (*p = 0.56*) (Fig. 2).

#### Serum C-reactive protein (CRP) values (mg/dl)

Serum CRP values gradually decreased compared to the baseline values in both groups during the treatment and the follow-up period, and the decrease in the study group (*p = 0.03*) was found more significant than the decrease in the control group (*p = 0.05*). In addition, CRP values ​​in the study group were found to be significantly lower than the control group at the end of the follow-up period (*p = 0.01*) (Fig. 2).

#### Serum ferritin values (mg/dl)

Serum ferritin values increased compared to the baseline values in both groups during the treatment period (*p = 0.06* and *p = 0.04* for study and control groups). Although in the study group, serum ferritin values started to decrease during the follow-up period, and showed a significant decrease compared to the baseline values (*p = 0.04*) at the end of the follow-up period; while it was found that it continued to increase compared to the baseline levels in the control group (*p = 0.01*). When the ferritin values of both groups were compared at the end of the follow-up period, it was found to be significantly higher in the control group (*p = 0.005*) (Fig. 2).

#### Serum D-dimer values (mg/L)

During the treatment period, serum D-dimer values ​​increased significantly in both groups (*p = 0.003* and *p = 0.02* for study and control groups) compared to the initial values. On the other hand, in the follow-up period, D-dimer values ​​started to decrease in both groups and at the end of the follow-up period, the values ​​in the study group reached a level significantly below the baseline values (*p = 0.04*), while the decrease in the control group was not found sufficient (*p = 0.11*) and was higher than the baseline values. At the end of the follow-up period, the difference between D-dimer levels in both groups was significant (*p = 0.03*) (Fig. 2).

### Genetic examination results and side effects

In our study, blood sample was taken with the first dose of ivermectin and haplotype analysis was performed in MDR-1/ABCB1 and CYP3A4 genes in the whole study group. In the light of the literature data, cases with mutations that has been reported to be able to reduce enzyme activity and cause the function losing (total 6 cases) were excluded from the study with the prediction that they were in the risk group in terms of developing complications, and ivermectin treatment was discontinued in these cases.

One of the ABCB1 (NM_000927.4)1236 T > C/2677 T > G/3435 T > C CGT, CTC, TTC alleles was detected in all 6 patients excluded from the study. CGC/CGT, CGC/CTC, CGC/TTC, TTT/CGT genotypes were observed only in those with complications. Since CGC and TTT alleles are the most frequently observed alleles that are also frequently observed in patients without complications, so the development of complications was thought to be related to CGT, CTC, TTC alleles.

In one of these 6 patients, S400I (c.1199G > A), CM068130, rs2229109 were additionally detected in the ABCB1 gene. Although there are data (PMID:16917872) indicating that the S400I change reduces membrane transport and therefore may cause drug resistance, no complications that we would consider to be due to ivermectin were observed in this patient.

Three of the 6 patients had agitation symptoms that disappeared on their own within one to 2 days. In two of these 3 patients, ABCB1 (NM_001348945.1):c.210G > A(p.Gly70=) genotype was additionally detected. Serious side effects such as delirium-like behavior, agitation, aggressive attitude and altered state of consciousness were observed in the remaining two of the 6 patients. In one of these two patients, side effects were controlled with haloperidol and the patient’s symptoms disappeared within 1 week. In the other patient, the side effects continued for 2 weeks, were controlled with remifentanil and dexmedetomidine and discharged with full recovery after being followed up in the hospital for 1 month. In this patient who has the most severe and prolonged symptoms, c.1191C > T (p.T363M) change was also detected in the CYP3A4 gene additionally.

No side effects or complications related to ivermectin were observed in patients other than those who were discontinued ivermectin at the first dose of treatment due to the risk of side effects according to genetic findings and excluded from the study.

When side effects of drugs other than ivermectin were evaluated in patients, it was found that two patients in the control group had nausea and vomiting, and one patient in the control group had a two-fold increase in ALT (alanine transaminase). None of these side effects were severe enough to require termination of treatment in patients.

## Discussion

COVID-19 disease caused by SARS CoV-2 causes severe viral pneumonia at rates varying between 7 and 14.8%, especially in some patients in the risk group. Its mortality is reported to be between 2 and 4%. Unfortunately, there are no proven treatments for patients with COVID-19 disease but drugs with antiviral, anti-inflammatory and immunomodulatory properties that are currently used in the treatment of COVID-19 [3, 10–14].

Advanced age (> 65), hypertension (HT) or the presence of coronary heart disease, diabetes mellitus (DM) and male gender are risk factors that have been shown to be associated with severe prognosis [12, 14, 15]. In accordance with the literature, the three most common comorbid conditions in severe COVID-19 patients were identified as HT, DM, and coronary artery disease in our study (Table 1). In our study, although hypertension was more common in the study group than in the control group, other comorbidities were higher in the control group. But we did not think that this difference would have a negative effect on the study results because it was not statistically significant. Our study is the first prospective randomized controlled trial investigating the efficacy of ivermectin in the treatment of patients with severe COVID-19.

In the literature, there are a few prospective randomized controlled studies evaluating the efficacy of hydroxychloroquine, lopinavir-ritonavir, remdesivir, and favipiravir drugs, which are among the treatment options of COVID-19 patients [16–19]. When these studies were examined, it was reported that remdesivir shortened the recovery time compared to placebo, and favipiravir increased viral clearance [18, 19].

In our study, we found that patients who added ivermectin to the HFA combination therapy (study group) had a higher rate of clinical improvement compared to patients who received only HFA combination therapy (control group). Similarly, at the end of the follow-up period, mortality rates were found to be lower in the study group, compared to the control group receiving only HFA combination therapy. Although clinical improvement and mortality differences between study and control group were not statistically significant, these differences can be more clearly revealed in new studies including larger patient series.

Considering that the patients included in our study have severe COVID-19, it can be thought that we have achieved a better clinical response with ivermectin treatment than the antiviral drugs studied so far. In a retrospective cohort analysis conducted in Florida, it was reported that mortality was reduced in COVID-19 patients with the use of a single dose of ivermectin, supporting our results [20]. Yet our results suggest that ivermectin may be an alternative or an additional option to standart treatment protocols in the treatment of COVID-19 disease.

SpO2 are below physiological levels in most patients who develop COVID-19 pneumonia and in all patients with severe prognosis. Also SpO2 levels cannot reach normal limits most of the time despite oxygen support or other supportive treatments in patients with severe prognosis. Increase in SpO2 level with treatment in patients is a significant indicator of clinical response to treatment [21, 22]. In our study, SpO2 levels increased compared to the baseline levels in both groups during the treatment and follow-up period, but reached the desired levels in the study group at the end of the follow-up period (95.4%) and were found to be significantly higher than the control group. Accordingly, it can be said that adding ivermectin to the treatment has a more positive effect on the treatment of Covid-19 pneumonia than the current treatment protocol.

As a matter of fact, in a study comparing the efficacy of single dose ivermectin + doxycycline combination and azithromycin + hydroxychloroquine combination therapies in patients with mild to moderate severity COVID-19, it was reported that symptomatic improvement was achieved in a shorter time with the combination containing ivermectin [23].

The best indicator of oxygenation in the blood is the PaO2/FiO2 ratio. Its normal range is 300–500 mmHg and being < 200 mmHg indicates severe hypoxemia. An increase in this rate indicates clinical improvement in severe COVID-19 patients [24]. We created the research universe from patients with severe COVID-19 at high mortality risk. Although the initial PaO2/FiO2 ratios ​​of the patients in the study group were lower than the control group and there was a slight decrease at the beginning of the treatment period, the fact that they reached the higher values ​​at the end of the treatment and follow-up period compared to the baseline levels and control group can be evaluated as an indicator of the effectiveness of adding ivermectin to the treatment. The fact that adequate response at PaO2/FiO2 ratios was obtained in the late periods of the study, suggests that more positive results can be obtained by starting ivermectin treatment earlier before severe pneumonia develops. The suggestion that ivermectin can be used in patients with mild or moderate COVID-19 pneumonia should be supported by further studies.

In COVID-19 disease, serum ferritin, CRP and D-dimer levels, blood lymphocyte count, and PNL/L ratio are laboratory parameters that have been shown to be associated with prognosis. It is reported that the prognosis is worse especially in patients whose lymphocyte count does not change despite the treatments given and whose ferritin and D-dimer values remain high. Therefore, changes in these parameters are considered as substantial indicators of clinical response in patients receiving treatment [25–28].

In our study, with the addition of ivermectin to the treatment, it was observed that a more pronounced and earlier increase in lymphocyte counts was achieved in patients in the study group compared to the control group. While PNL/L ratio, one of the prognosis indicators, started to decrease early in the treatment period in the study group, it increased in the control group. In the study group, this decrease continued significantly in the follow-up period. But in the control group, a decrease in the PNL/L ratio was observed only towards the middle of the follow-up period. This result shows that ivermectin provides earlier treatment efficacy in the treatment of COVID-19 infection compared to existing protocols.

In the literature, it has been reported that the prognosis will be poor in patients > 50 years of age and with PNL/L > 3.13 and intensive care follow-up is required [27]. Therefore, the early decrease provided by ivermectin in PNL/L ratios can contribute to shortening the intensive care period and improving the prognosis in COVID-19 infections. At the end of the follow-up period, it was observed that PNL/L ratios were lower in both groups compared to the baseline values, the decrease in the study group was more pronounced than the control group, but there was no significant difference between both groups. While the decrease in the PNL/L ratio continued significantly in the study group until the 3rd day in the follow-up period, there was a slight increase on the 5th day. The reason for this may be leukocytosis due to secondary bacterial infections (unspecified data) that we detected in patients.

The fact that serum CRP and D-dimer values decreased significantly earlier and faster in the study group, and serum ferritin values decreased significantly in the study group while continued to increased in the control group, can be considered as an indicator that adding ivermectin increases the effectiveness of the severe Covid-19 infection treatment.

When the results of these five laboratory parameters which are valuable in the follow-up of the prognosis of the disease (blood lymphocyte count, serum ferritin, CRP, D-dimer levels and PNL/L ratio) were evaluated; it was found that ivermectin was effective in the treatment of COVID-19, it seems to provide an earlier treatment response and supports the idea that ivermectin or adding ivermectin to current treatment protocols may be an option for the treatment of COVID-19.

In our study, no different side effects were observed in patients receiving ivermectin compared to patients receiving standard therapy. However, three of the 6 patients with MDR-1/ABCB1 or CYP3A4 gene mutation who received the first dose of ivermectin had mild (agitation) and two had severe side effects (agitation, delirium-like behavior, aggressive behavior and consciousness changes).

The determination of ABCB1 (NM_000927.4)1236 T > C/2677 T > G/3435 T > C genotypes is important in determining the risk of side effects in drug use. ABCB1 (NM_000927.4)1236 T > C/2677 T > G/3435 T > C genotype was detected in all 6 patients who excluded from the study. Therefore, this haplotype including in cases where it is heterozygous, was considered as the main haplotype in terms of complication development, and at the end of the study it was determined that this prediction was mostly correct.

In one of these 6 patients, S400I (c.1199G > A), CM068130, rs2229109 genotype were found in addition to ABCB1 gene. Although it has been stated in the literature that the S400I change may alter membrane transport and cause drug resistance (PMID: 16917872), no side effects related to ivermectin developed in this patient.

ABCB1 (NM_001348945.1): c.210G > A(p.Gly70 =) genotype was additionally found in two of our 3 patients with mild side effects. On the other hand, the detection of the same genotype in 2 of 31 patients without side effects suggests that this genotype change has no effect on ivermectin metabolism.

In our patient, who developed the most severe and longest lasting side effect associated with ivermectin, in addition to ABCB1 mutation, a change in CYP3A4 gene was found to be c.1191C > T(p.T363M). It has been reported in the literature that the T363M change detected in the CYP3A4 gene reduces the function of the enzyme. Therefore, it has been recommended in the literature to reduce the drug dose (HGMD: CM015322). After the first dose of ivermectin, agitation, delirium-like symptoms, aggression and changes in consciousness were observed in this patient who was given remifentanyl and dexmedetomidine for sedation and was excluded from the study. Midazolam administration was also required and it took about 2 weeks for symptoms to disappear in this patient. The reason for the longer and more severe clinical symptoms in this patient compared to the patients with other drug side effects was considered to be the coexistence of both ABCB1 and CYP3A4 changes. This finding suggests that the CYP3A4 gene is also effective and important in ivermectin metabolism.

In our study, in patients who developed side effects due to ivermectin, symptoms disappeared completely within 2 weeks in 2 patients with severe side effects and in 1–2 days in 3 patients with mild side effects. All these results suggest that, the drug can be used safely in patients who do not have a mutation that may affect ivermectin metabolism. If it is decided to use drug at the community level or in large groups, since sequence analysis is not possible in practice due to time constraints, patients should be followed up closely in terms of encephalopathy-like symptoms affecting the central nervous system, and symptoms can be controlled in these patients with appropriate treatment and follow-up.

Our study is the first randomized controlled prospective study in the literature in which MDR-1/ABCB1 and CYP3A4 gene variants that may cause changes in ivermectin dose were investigated in patients with COVID-19. There are warnings in the literature as the study of Caly et al., about the possible toxic effects of Ivermectin that is a promising drug for the treatment of COVID-19 and the FDA also draws particular attention to this issue [7, 29]. However, our result sheds light on the concerns in this regard.

One limitation of our study is that the interactions of the drugs used were not evaluated. However, we think that there is no adverse drug interaction due to the absence of any laboratory changes that cannot be explained with the clinical conditions of the patients [30].

## Conclusion

This study suggests that ivermectin may be an alternative drug that can be used in the treatment of COVID-19 disease or an additional option to current treatment protocols. Even when used in severe COVID-19 patients, it can provide an increase in clinical recovery, improvement in prognostic laboratory parameters, and a decrease in mortality rates. It is predicted that ivermectin can be used safely without causing any serious side effects in patients without MDR-1/ABCB1 and/or CYP3A4 gene mutation, and the emerging side effects can be eliminated with appropriate treatment. All these results suggest that ivermectin may be a hope in the treatment of COVID-19 disease and these results we achieved in our study should be supported with further studies, especially with more cases including early stage COVID-19 patients.

## Supplementary Information

**Additional file 1: Supplementary Table 1.** Laboratory parameter changes.

## Data Availability

The anonymised datasets used and/or analysed during the current study are available from the corresponding author on reasonable request.
