# Supplementary material for: Evaluation of the effectiveness and safety of adding ivermectin to treatment in severe COVID-19 patients
Source: BMC Infect Dis. 2021 May 4;21:411. doi: 10.1186/s12879-021-06104-9 (PMC8093585; doi:10.1186/s12879-021-06104-9)
Supplement: Supplementary file 1 — Additional file 1: Supplementary Table 1. Laboratory parameter changes. [file 12879_2021_6104_MOESM1_ESM.docx]

**Supplementary Table 1: Laboratory parameter changes**

| **Days** | **Groups** | **SpO2** | ***p*** | **PaO2/FiO2** | ***p*** | **Lymphocyte count** | ***p*** | **N/L ratio** | ***p*** | **CRP** | ***p*** | **Ferritin** | ***p*** | **D-dimer** | ***p*** |
| --- | --- | --- | --- | --- | --- | --- | --- | --- | --- | --- | --- | --- | --- | --- | --- |
| **Baseline** | Study | 89.93±6.51 | ***0.64*** | 158.83±88.15 | ***0.15*** | 932±483 | ***0.42*** | 8.77±8.35 | **0.79** | 34.03±132.49 | ***0.26*** | 682.75±470.08 | ***0.35*** | 1.25±1.71 | ***0.98*** |
|  | Control | 89.67±5.09 |  | 197.44±102.31 |  | 1010±438 |  | 7.48±6.41 |  | 21.50±43.03 |  | 747.05±800.54 |  | 1.32±2.04 |  |
| **TD1**  **(1st day)** | Study | 92.85±4.86 |  | 147.31±74.15 |  | 928±607 |  | 10.82±8.55 |  | 17.06±38.93 |  | 834.94±624.12 |  | 1.40±1.73 |  |
|  | Control | 90.50±7.47 |  | 181.83±99.62 |  | 1034±450 |  | 7.74±7.47 |  | 22.31±42.87 |  | 783.03±827.10 |  | 2.80±5.66 |  |
| **TD3**  **(3rd day)** | Study | 93.07±4.12 |  | 147.74±83.30 |  | 1021±648 |  | 9.02±13.08 |  | 9.91±15.31 |  | 875.90±694.52 |  | 3.24±11.60 |  |
|  | Control | 91.90±4.97 |  | 174.77±94.74 |  | 977±575 |  | 9.26±7.58 |  | 24.40±45.67 |  | 881.17±779.95 |  | 4.14±9.91 |  |
| **TD5**  **(5th day)** | Study | *93.52*±4.36 | ***0.14*** | *178.94*±98.21 | ***0.68*** | *1273*±822 | ***0.15*** | *7.16*±4.97 | ***0.37*** | *5.18*±5.45 | ***0.002*** | *875.12*±1193.06 | ***0.12*** | *5.85*±5.28 | ***0.22*** |
|  | Control | *93.00*±3.25 |  | *180.13*±95.43 |  | *968*±477 |  | *9.88*±8.45 |  | *19.43*±30.71 |  | *1028.24*±777.08 |  | *3.58*±8.27 |  |
| **Primary**  **End-point**  ***P value*** | Study | ***0.005*** |  | ***0.000*** |  | ***0.01*** |  | ***0.41*** |  | ***0.006*** |  | ***0.06*** |  | ***0.003*** |  |
|  | Control | ***0.003*** |  | ***0.07*** |  | ***0.50*** |  | ***0.42*** |  | ***0.82*** |  | ***0.038*** |  | ***0.02*** |  |
| **FD1**  **(6th day)** | Study | 94.54±2.21 |  | 199.83±85.02 |  | 1403±869 |  | 6.90±11.84 |  | 4.61±6.40 |  | 628.45±580.10 |  | 1.37±2.53 |  |
|  | Control | 92.43±2.86 |  | 204.28±109.51 |  | 916±411 |  | 10.49±7.10 |  | 25.84±47.59 |  | 1076.88±704.05 |  | 3.45±6.60 |  |
| **FD3**  **(8th day)** | Study | 94.24±2.76 |  | 227.43±103.71 |  | 1668±819 |  | 5.81±9.99 |  | 3.47±5.20 |  | 433.48±641.82 |  | 0.89±2.45 |  |
|  | Control | 92.91±2.71 |  | 211.75±127.62 |  | 1086±880 |  | 9.66±10.99 |  | 19.08±33.09 |  | 1097.57±595.22 |  | 1.63±1.38 |  |
| **FD5**  **(10th day)** | Study | *95.35*±2.72 | ***0.03*** | *236.33*±85.66 | ***0.39*** | *1698*±1438 | ***0.24*** | *7.34*±8.09 | ***0.56*** | *3.61*±4.60 | ***0.02*** | *494.71*±349.78 | ***0.005*** | *0.71*±0.96 | ***0.03*** |
|  | Control | *93.00*±3.93 |  | *220.78*±127.26 |  | *1256*±710 |  | *6.19*±4.85 |  | *9.24*±10.77 |  | *1206.90*±782.84 |  | *1.49*±2.28 |  |
| **Secondary**  **End-point**  ***p value*** | Study | ***0.011*** |  | ***0.008*** |  | ***0.008*** |  | ***0.104*** |  | ***0.004*** |  | ***0.042*** |  | ***0.044*** |  |
|  | Control | ***0.004*** |  | ***0.72*** |  | ***0.05*** |  | ***0.69*** |  | ***0.01*** |  | ***0.01*** |  | ***0.11*** |  |

***TD: Treatment Day, FD: Follow-up Day, SpO2: Peripheral Capillary Oxygen saturation, PaO2: Partial Pressure of Oxygen saturation, N/L: Neutrophil/Lymphocyte, CRP: C-Reactive Protein***
